# Involvement of Calpain/p35-p25/Cdk5/NMDAR Signaling Pathway in Glutamate-Induced Neurotoxicity in Cultured Rat Retinal Neurons

**DOI:** 10.1371/journal.pone.0042318

**Published:** 2012-08-01

**Authors:** Yanying Miao, Ling-Dan Dong, Jie Chen, Xiao-Chen Hu, Xiong-Li Yang, Zhongfeng Wang

**Affiliations:** Institutes of Brain Science, Institute of Neurobiology and State Key Laboratory of Medical Neurobiology, Fudan University, Shanghai, China; Universidad de Castilla-La Mancha, Spain

## Abstract

We investigated possible involvement of a calpain/p35-p25/cyclin-dependent kinase 5 (Cdk5) signaling pathway in modifying NMDA receptors (NMDARs) in glutamate-induced injury of cultured rat retinal neurons. Glutamate treatment decreased cell viability and induced cell apoptosis, which was accompanied by an increase in Cdk5 and p-Cdk5^T15^ protein levels. The Cdk5 inhibitor roscovitine rescued the cell viability and inhibited the cell apoptosis. In addition, the protein levels of both calpain 2 and calpain-specific alpha-spectrin breakdown products (SBDPs), which are both Ca^2+^-dependent, were elevated in glutamate-induced cell injury. The protein levels of Cdk5, p-Cdk5^T15^, calpain 2 and SBDPs tended to decline with glutamate treatments of more than 9 h. Furthermore, the elevation of SBDPs was attenuated by either D-APV, a NMDAR antagonist, or CNQX, a non-NMDAR antagonist, but was hardly changed by the inhibitors of intracellular calcium stores dantrolene and xestospongin. Moreover, the Cdk5 co-activator p35 was significantly up-regulated, whereas its cleaved product p25 expression showed a transient increase. Glutamate treatment for less than 9 h also considerably enhanced the ratio of the Cdk5-phosphorylated NMDAR subunit NR2A at Ser1232 site (p-NR2A^S1232^) and NR2A (p-NR2A^S1232^/NR2A), and caused a translocation of p-NR2A^S1232^ from the cytosol to the plasma membrane. The enhanced p-NR2A^S1232^ was inhibited by roscovitine, but augmented by over-expression of Cdk5. Calcium imaging experiments further showed that intracellular Ca^2+^ concentrations ([Ca^2+^]_i_) of retinal cells were steadily increased following glutamate treatments of 2 h, 6 h and 9 h. All these results suggest that the activation of the calpain/p35-p25/Cdk5 signaling pathway may contribute to glutamate neurotoxicity in the retina by up-regulating p-NR2A^S1232^ expression.

## Introduction

Glutamate, a major excitatory neurotransmitter in the central nervous system (CNS), plays crucial roles in many physiological functions through activating its ionotropic and/or metabotropic receptors [1], [2]. Meanwhile, extracellular excessive glutamate or prolonged exposure to glutamate may cause neuronal dysfunction and even degeneration, an effect that refers to as glutamate neurotoxicity [3], [4]. Excessive Ca^2+^ influx through glutamate receptors is associated with glutamate neurotoxicity, which leads to an activation of enzymes for degrading of proteins, membranes and nucleic acids [5]. Glutamate neurotoxicity has been implicated in a variety of acute and chronic CNS disorders, as well as many forms of retinal injury and disease, such as ischemia, diabetic retinopathy and glaucoma [6]–[13]. Whilst the mechanisms underlying glutamate neurotoxicity are complex, NMDA receptor (NMDAR)- and non-NMDAR-mediated Ca^2+^ overload may be a key factor [13]–[16].

In the retina, NMDARs are widely expressed in neuronal cells [17] and these receptors are involved in glutamate-induced apoptotic death of retinal neurons [18], [19]. For example, a prolonged injection of glutamate of low-concentration induces rat retinal ganglion cell (RGC) death [20]. On the other hand, administration of the NMDA channel blocker MK-801/memantine prevents RGC death in rat experimental glaucoma models, retinal ischemia and diabetic retinopathy [18], [21]–[24], and reduces the expression of pre-apoptosis molecules in rat retinal transient ischemia [25]. Nevertheless, what are precise intracellular signaling pathways for retinal glutamate neurotoxicity and how NMDARs are changed in intrinsic properties need to be further explored. Moreover, excessive stimulation of non-NMDARs, which are abundantly distributed in the retina, has been recently shown to play a crucial role in glutamate neurotoxicity as well [15], [16].

Cyclin-dependent kinase 5 (Cdk5), along with its neuron-specific activating cofactors p35 and p39 [26], [27], plays multiple roles in neuronal development and synaptic plasticity [28]–[32]. Furthermore, p35 may be proteolytically cleaved to p25 by the Ca^2+^-dependent protease calpain [33]–[35]. It is known that Cdk5/p35 is implicated in many neurological disorders [36]–[44]. Specifically, elevated phosphorylation of the NMDAR subunit NR2A at Ser1232 (p-NR2A^S1232^) by Cdk5/p35 may contribute to ischemic rat hippocampal CA1 neuron death [45] and to RGC apoptotic death in a rat experimental glaucoma model [44]. The effects caused by the activation of Cdk5 may be mediated by different signaling pathways [37], [46]–[49]. In the present work we demonstrated the involvement of a distinct calpain/p35-p25/Cdk5/NMDAR signaling pathway in glutamate-induced injury of primary cultured rat retinal neurons.

## Materials and Methods

### Primary retinal neuronal culture and transfection

All experimental procedures described here were carried out in accordance with the National Institutes of Health (NIH) guidelines for the Care and Use of Laboratory Animals and the guidelines of Fudan University on the Ethical Use of Animals. And all animal care and procedures in the present experiments were approved by the Institutes of Brain Science, Institute of Neurobiology and State Key Laboratory of Medical Neurobiology of Fudan University, Shanghai, China. During this study all efforts were made to minimize the number of animals used and their suffering. Primary retinal neuronal cultures were prepared as described previously by Kerrison and Zack [50] with minor modification. Briefly, retinas of newborn Sprague-Dawley rats (2 d old), obtained from SLAC Laboratory Animal Co. Ltd (Shanghai, China), were removed after anesthesia and digested by trypsinization (0.25% for 15 min at 37°C). Retinal neurons were mechanically dissociated by using a fire-polished Pasteur pipette. The cell suspension was plated at a density of 1.2×10^6^ onto poly-D-lysine-coated 35 mm dishes and cultured in a neurobasal medium (Gibco BRL, Life Technologies, Rockville, MD, USA), supplemented with 2% B27 and 2 mM glutamine, in a humidified 5% CO_2_ incubator at 37°C. In the CNS, under these culture conditions, more than 90% of cells in the cultures were neuronal cells [51]. MAP-2, a neuronal marker, was used to label retinal neurons. RGCs were identified by using specific cell markers, Thy-1.1 and Brn-3a [52], [53]. Experiments were performed on the 8^th^ day of neurons in culture.

For cell transfection experiments, the cells were seeded at 6-well plate and transfected with 1 µg pcDNA3-Cdk5GFP plasmid DNA (Addgene plasmid 1346, kindly provided by Dr. Li-huei Tsai) or empty vector, according to the method of Jiang and Chen [54], with the CalPhos™ mammalian transfection kit (Clontech Laboratories, Inc. CA, USA). Transfection rate was about 40% at 24 h after being transfected.

### Assessment of retinal neuron viability

Cell viability was examined by the 3-(4, 5-dimethylthiazole-2-yl)-2, 5 –dipenyltetrazolium bromide (MTT) assay. The cells were seeded at a density of 2×10^5^ per well onto poly-D-lysine-coated 96-well plates. Retinal neurons were treated by glutamate of various concentrations for 24 h or by 0.5 mM glutamate for different time periods. Roscovitine (5 and 25 µM) (Biomol, Plymouth Meeting, PA, USA), a Cdk5 inhibitor, was added to the medium 30 min before glutamate treatment (GT). MTT was added to the medium with a final concentration of 0.5 mg/ml 4 h before the end of the experiments. The supernatant was removed and replaced by 150 µl of dimethyl sulfoxide (DMSO), and the cell viability was measured on a microplate reader at 550 nm. Experiments were repeated independently three times in triplicate and data were represented as MTT reductions relative to control.

### Flow cytometry

Flow cytometry was employed to identify RGCs and apoptotic cells in the culture. The cultured cells were washed twice with phosphate-buffered saline (PBS) and detached with 0.05% trypsin–EDTA for 3–5 min at room temperature. The cells were collected by centrifugation and washed with flow cytometry buffer consisting of 2% bovine serum albumin (BSA) in PBS, and were then incubated with phycoerythrin (PE)-conjugated monoclonal antibody against Thy1.1 (Abcam, Cambridge, MA, USA) for 30 min. The cells were re-washed with flow cytometry buffer again and pelleted, and then re-suspended in flow cytometry buffer. Non-specific fluorescence was determined using equal aliquots of the cell preparation that were incubated with anti-mouse monoclonal antibodies. Data were acquired and analyzed on FACSCalibur with CellQuest software (Becton–Dickinson, Germany).

Neuronal apoptosis was assayed by Annexin V-FITC apoptosis detection kit (BD Biosciences Pharmingen, San Diego, CA, USA) according to the manufacturer's protocol. Briefly, cultured neurons were incubated with 5 µl of Annexin V-FITC and 5 µl propidium iodide at room temperature for 5 min in the dark, and stained cells were analyzed by flow cytometry. For analysis of apoptotic RGCs, Annexin V-FITC and propidium iodide were added to flow cytometry buffer 5 min before the end of PE-Thy1.1 staining, as described above.

### Western blot analysis

Western blot analysis was conducted as previously described [44], [55], with some modifications. The cells were collected and lysed in lysis buffer (50 mM Tris-Cl, 150 mM NaCl, 1% Triton X-100, 0.1% aprotinin, 1 mM phenylmethylsulfonyl fluoride, 1 mM sodium orthovanadate, and 25 mM sodium fluoride, pH 7.4). The concentrations of total proteins were measured using a standard bicinchoninic acid (BCA) assay kit (Pierce Biotechnology, Rockford, IL, USA). The samples were denatured by boiling for 5 min. The proteins in 10 µg samples were separated by 8%,10% or 15% SDS-PAGE gel, and then electrotransferred to a PVDF membrane (Immobilon-P, Millipore, Billerica, MA, USA) using Mini-PROTEAN 3 Electrophoresis System and Mini Trans-Blot Electrophoretic Transfer System (Bio-Rad, Hercules, CA, USA). The blots were blocked with a buffer containing 0.05% Tween-20 and 5% defatted milk, and then treated sequentially with primary antibodies at 4°C overnight and secondary antibodies for 1 h at room temperature. Primary antibodies used in the present work were p-NR2A^S1232^ antibody (#2056, 1∶500, Tocris Bioscience, MO, USA), NR2A antibody (#320600, 1∶500, Invitrogen, Carlsbad, CA, USA), Cdk5 antibody (#20502, clone DC17, 1∶1000, Millipore, Billerica, MA, USA), calpain 2 antibody (#2539, 1∶1000, Cell Signal Technology, MA, USA), spectrin alpha II antibody (sc-46696, 1∶1000; Santa Cruz Biotechnology, CA, USA), p-Cdk5^T15^ (sc-12918, 1∶1000; Santa Cruz Biotechnology), p35/p25 antibody (sc-820, 1∶1000; Santa Cruz Biotechnology) and β-actin antibody (A3853, 1∶2000, Sigma, Saint Louis, MO, USA). The blots were incubated with chemofluorescent reagent (Pierce Co., Rockford, IL, USA) followed by exposing to X-ray film in a dark room. For sequential immunoblotting, the blots were re-blocked, tested for residual signal and then stripped with restore Western blot stripping buffer (Pierce Co., Rockford, IL, USA) if necessary. Experiments were performed in triplicate. The protein bands were quantitatively analyzed with NIH Image Analysis software.

### Preparation of plasma membrane and cytosolic fractions

Plasma membrane and cytosolic fractions were prepared using the Nucl-Cyto-Mem preparation kit (Applygen Technologies Inc, Beijing, China) following the manufacturer's protocol. Briefly, the cells were harvested with 0.25% trypsinization at a density of 1×10^7^, and lysed in cytosol extraction reagent provided by the kit. Complete cell disruption was done by using a 25-gauge needle and a syringe for 15 strokes. The homogenates were centrifuged at 800×*g* for 5 min at 4°C. The supernatant was added to the membrane extraction reagent and centrifuged 14,000×*g* for 30 min at 4°C to obtain crude membrane pellets. The obtained supernatant was cytosolic fraction, which was then suspended in SDS sample buffer and analyzed by immunoblotting as described above. The same volume of samples was loaded in 8% SDS-PAGE to assay p-NR2A^S1232^ expression.

### Immunofluorescent staining

Immunofluorescent staining was performed following the procedure described in detail previously [55], [56]. Neurons grown on cover slips were fixed with 4% paraformaldehyde in PBS. After washing with PBS, the cells were incubated in 3% BSA and 0.1% Triton X-100 in PBS for 30 min and incubated with anti-p-NR2A^S1232^ antibody (#2056, 1∶100, Tocris Bioscience, Missouri, USA), anti-Brn-3a antibody (sc-8429, 1∶1000, Santa Cluz, Biotechnology, CA, USA) or anti-MAP-2 antibody (1∶1000, Sigma, St Louis, MO, USA) at 4°C overnight. F(ab′)2 fragment–Cy3 conjugated anti-mouse IgG (1∶200, Sigma, St Louis, MO, USA) was used as the secondary antibody. After washing, the samples were mounted with anti-fade mounting medium with DAPI (Vector Laboratories, Burlingame, CA, USA) and examined using a Leica SP2 confocal laser scanning microscope at a 40× oil-immersion objective lens. All experiments were performed at least in triplicate.

### Calcium imaging

Changes in intracellular calcium concentrations ([Ca^2+^]_i_) of cultured retinal neurons, which were grown on cover slips and treated with glutamate (0.5 mM) for different time periods (2 h, 6 h, 9 h, 12 h), were assessed using the ratiometric dye Fura-2 AM (Dojindo, Kumamoto, Japan). Fura-2 AM, dissolved in DMSO, was added to the culture medium 30 min before the end of an experiment, with a final concentration being 5 µM. After rinsing with the culture medium twice, the Fura-2-loaded cultures were placed on the stage of an inverted fluorescence microscope (DMI 3000B; Leica) and perfused continually with bath solution (in mM): 25 HEPES, 128 NaCl, 5 KCl, 1 MgCl_2_, 2 CaCl_2_, and 30 glucose, pH 7.3. Digital fluorescent images at both wavelengths (340 and 380 nm excitation) were captured with a CoolSNAP HQ^2^ system (Photometrics, USA) at room temperature through a ×20/0.5 objective lens and an emission filter (510–550 nm). Three batches of retinal cell cultures were used for GT of each time period, and two cell dishes from each batch were selected. Five fields were randomly captured for each dish under microscope for the analysis. Therefore, total 30 digital fluorescent images were obtained for GT of each time period. In each image about 30–50 cells were randomly selected for calculating the ration (F340/F380) that is proportional to [Ca^2+^]_i_ of the cell under study, using MetaFluor Analyst software (Version 1.0.93, Universal Imaging Co., USA). The bath ground level of fluorescence (due to autofluorescence and camera noise), determined before the experiment, was then subtracted from all the data obtained. All averaged data of [Ca^2+^]_i_ were normalized to control, which are shown in pseudocolor.

For experiments, in which immunostaining with MAP-2 and Brn-3a was conducted following calcium imaging, Fluo-4 (5 µM), another fluorescent calcium indicator, was added to the culture medium for 30 min before the end of an experiment. And the cultures were observed under a Leica SP2 confocal laser scanning microscope.

### Statistical Analysis

Data, expressed as mean ± SEM, were analyzed using GraphPad Prism software (version 4.00). One-way ANOVA with Bonferroni's post test (multiple comparisons) was used with *p*<0.05 being considered significant.

## Results

### Glutamate-induced retinal neuron injury

**Figure 1:**
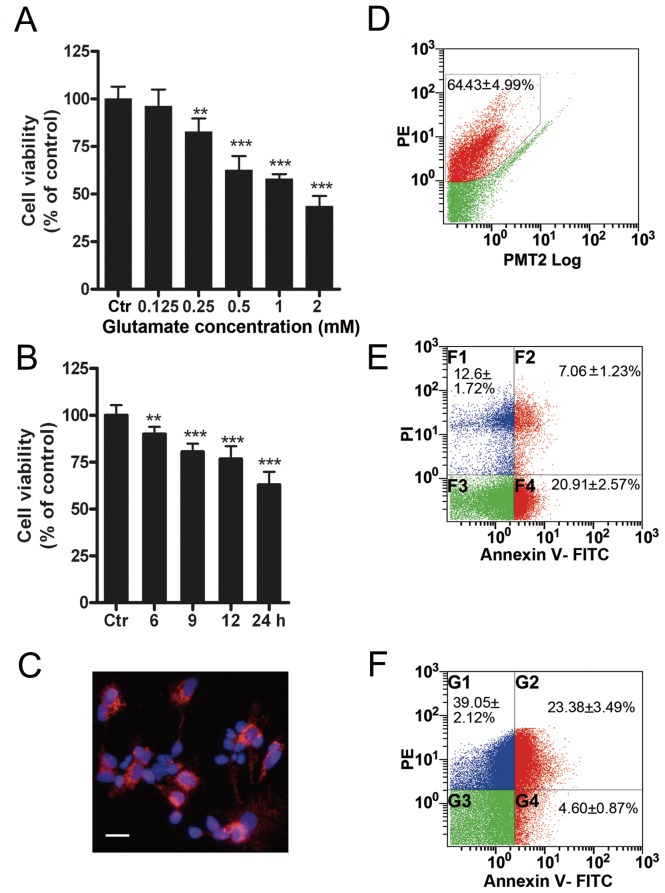
Glutamate-induced injury in cultured rat retinal neurons. (A) Glutamate treatment for 24 h [GT (24 h)] decreased the cell viability in a concentration-dependent manner. Cell viability was assayed by MTT method. Data are normalized to control (Ctr). n = 9 for each group, ** *p*<0.01, *** *p*<0.001 *vs* Ctr, one-way ANOVA. (B) Cell viability was decreased in a time-dependent manner following 0.5 mM GTs (6 h, 9 h, 12 h, 24 h), assayed by MTT method. n = 9 for each group, ** *p*<0.01, *** *p*<0.001 *vs* Ctr, one-way ANOVA. (C) Retinal ganglion cells (RGCs) were immunostained with Brn-3a antibody. Scale bar: 20 µm. (D) Flow cytometry analysis shows Thy1.1-positive RGCs (red, in the black polygon) in cultured retinal cells. n = 6. (E) Annexin V-FITC flow cytometry shows cell apoptosis (red) in cultured retinal cells after GT (24 h). F2 and F4 are the apoptotic cells at early and late phases respectively. n = 6. (F) Annexin V-FITC and PE-Thy1.1 flow cytometry shows cell apoptosis (red) of the PE-Thy1.1 staining RGCs. G2 and G4 are the apoptotic RGCs at early and late phases respectively. n = 6.

We evaluated GT-induced retinal cell injury using MTT assay. Fig. 1A shows the cell viabilities when cultured cells were treated with glutamate at increasing concentrations for 24 h. The cell viability was not much changed (96.1±8.9% of control) at 0.125 mM glutamate (n = 9, *p*>0.05). When the concentration of glutamate was higher than 0.25 mM, the cell viability was decreased in a dose-dependent manner [80.6±8.1% for 0.25 mM (n = 9, *p*<0.01), 62.6±7.3% for 0.5 mM (n = 9, *p*<0.001), 57.9±2.5% for 1 mM (n = 9, *p*<0.001) and 42.6±6.2% for 2 mM (n = 9, *p*<0.001) of control, respectively]. The bar chart in Fig. 1B shows the changes in cell viability with 0.5 mM GT of different time periods. The cell viability showed a steady decrease with GT of increasing times [89.9±3.9% at 6 h (n = 9, *P*<0.01), 80.5±4.3% at 9 h (n = 9, *P*<0.001), 76.7±6.8% at 12 h, n = 9, *P*<0.001), 62.9±6.9% at 24 h, n = 9, *P*<0.001) of control]. In the experiments to follow, unless specified otherwise, 0.5 mM glutamate was used to treat cells for 24 h [GT (24 h)] as a standard protocol. To determine the ratio of RGCs in our culture conditions, these cells were either labeled by Brn-3a (Fig. 1C) and counted under a fluorescence microscopy, or by Thy1.1 immunostaining (Fig. 1D) and then measured by flow cytometry. The average number of Brn-3a-labeled cells was 57.6±3.8% of total cells (n = 3), which was very close to that of Thy1.1-positive cells (64.4±5.0%, n = 6). Following GT (24 h), 28.0±2.5% (n = 6) of cultured cells underwent apoptosis, including those at the apoptotic early phase (20.9±2.6%) and the late phase (7.1±1.2%) (Fig. 1E). A further analysis revealed that 23.4±3.5% (n = 6) of Thy1.1-positive RGCs were apoptotic ones (Fig. 1F). In other words, 83.6% (23.4/28.0) of the apoptotic cells were Thy1.1-positive RGCs.

### Involvement of activated Cdk5 in glutamate neurotoxicity

**Figure 2:**
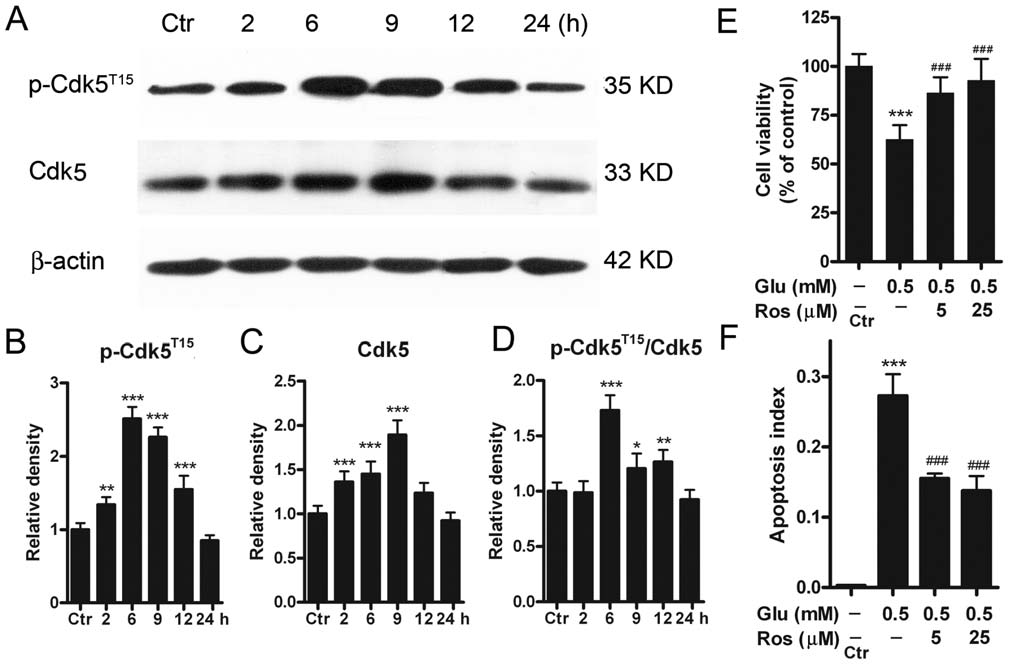
Protein levels of Cdk5 and p-Cdk5^T15^ in cultured rat retinal neurons following GTs. (A) Representative immunoblots showing the changes in Cdk5 and p-Cdk5^T15^ levels in cell extracts obtained from normal (Ctr) and glutamate-treated (0.5 mM for 2, 6, 9, 12 and 24 h) groups. (B, C, D) Bar charts summarizing the average densitometric quantification of immunoreactive bands of p-Cdk5^T15^ (B), Cdk5 (C) and ratios p-Cdk5^T15^/Cdk5 (D) in Ctr and glutamate-treated groups, respectively. All data are normalized to their corresponding β-actin data and then to Ctr. n = 6, * *p*<0.05, ** *p*<0.01 and *** *p*<0.001 *vs* Ctr, one-way ANOVA. (E) Bar chart summarizing the effects of roscovitine (Ros) on glutamate (Glu)-induced cell injury assayed by MTT method. Retinal neurons were incubated with Ros (5 and 25 µM) 30 min prior to a 24 h Glu (0.5 mM) exposure. Note that Ros rescued the Glu-induced decrease of cell viability. n = 5–9, *** *p*<0.001 *vs* Ctr; ^###^ *p*<0.001 *vs* data obtained in the absence of Ros, one-way ANOVA. (F) Bar chart summarizing the effects of Ros on Glu-induced apoptosis assayed by Annexin V-FITC flow cytometry. Retinal neurons were incubated with Ros (5 and 25 µM) 30 min prior to a 24 h Glu (0.5 mM) exposure. n = 6 for each group, *** *p*<0.001 *vs* Ctr; ^###^ *p*<0.001 *vs* data obtained in the absence of Ros, one-way ANOVA.

Cdk5 is involved in ischemic hippocampal CA1 cell death [45] and RGC apoptotic death in glaucoma rats [44]. It has been shown that phosphorylation of Cdk5 at the site of tyrosine 15 (p-Cdk5^T15^) increases the enzymatic activity of Cdk5/p35 complex in a variety of neurons [57]–[60]. We examined whether the protein levels of Cdk5 and p-Cdk5^T15^ may be changed in retinal neurons following GTs. In these experiments cultured cells were treated with 0.5 mM glutamate for 2, 6, 9, 12, and 24 h respectively. Fig. 2A shows representative results obtained by Western blotting, presenting the expression levels of Cdk5 and p-Cdk5^T15^ following GTs of different periods of time. As shown in Fig. 2B, the average p-Cdk5^T15^ level was steadily increased with GTs (≤9 h) [134.1±10.1% with GT (2 h), n = 6, *p*<0.01), 251.0±16.4% with GT (6 h) (n = 6, *p*<0.001), 225.9±13.6% of control with GT (9 h) (n = 6, *p*<0.001)] and then tended to decline with GT (12 h) [154.8±18.6% of control (n = 6, *p*<0.001)]. With GT (24 h) it was comparable to the control level (85.1±7.1% of control, n = 6, *p*>0.05) (Fig. 2B). The Cdk5 protein level showed a similar change (Fig. 2C). It was steadily increased to 136.0±11.8% with GT (2 h) (n = 6, *p*<0.001), 145.1±14.2% with GT (6 h) (n = 6, *p*<0.001), 186.7±16.5% of control with GT (9 h) (n = 6, *p*<0.001), and then decreased to 122.4±11.6% of control with GT (12 h) (n = 6, *p*>0.05). It returned to the control level following GT (24 h) (92.2±9.3% of control, n = 6, *p*>0.05). The ratio p-Cdk5^T15^/Cdk5 was increased to 173.0±13.8% (n = 6, *p*<0.001), 121.0±13.4% (n = 6, *p*<0.05), 126.5±10.8% of control (n = 6, *p*<0.01) following GTs (6 h, 9 h, 12 h) respectively, and then returned to the control level with GT (24 h) (92.3±8.9%, n = 6, *p*>0.05) (Fig. 2D).

To determine whether activated Cdk5 contributed to GT-induced retinal cell injury, the Cdk5 inhibitor roscovitine was added to the culture medium 30 min prior to GT (24 h). As shown in Fig. 2E, roscovitine of 5 µM increased the cell viability from 62.6±7.4% (n = 6) of control, obtained with GT alone but no roscovitine, to 88.1±8.9% (n = 6, *p*<0.001). There was no further increase in cell viability with 25 µM roscovitine (92.9±11.6% of control, n = 6). Consistently, roscovitine decreased the apoptosis index to15.4±0.7% (n = 6, *p*<0.001) from 27.3±3.1% (n = 6), obtained with GT alone (Fig. 2F). Again, no further decrease in apoptosis index was seen when the concentration of roscovitine was increased to 25 µM (13.2±2.1% of control, n = 6).

### Changes in protein levels of calpain 2, p35 and/or p25 in glutamate neurotoxicity

**Figure 3:**
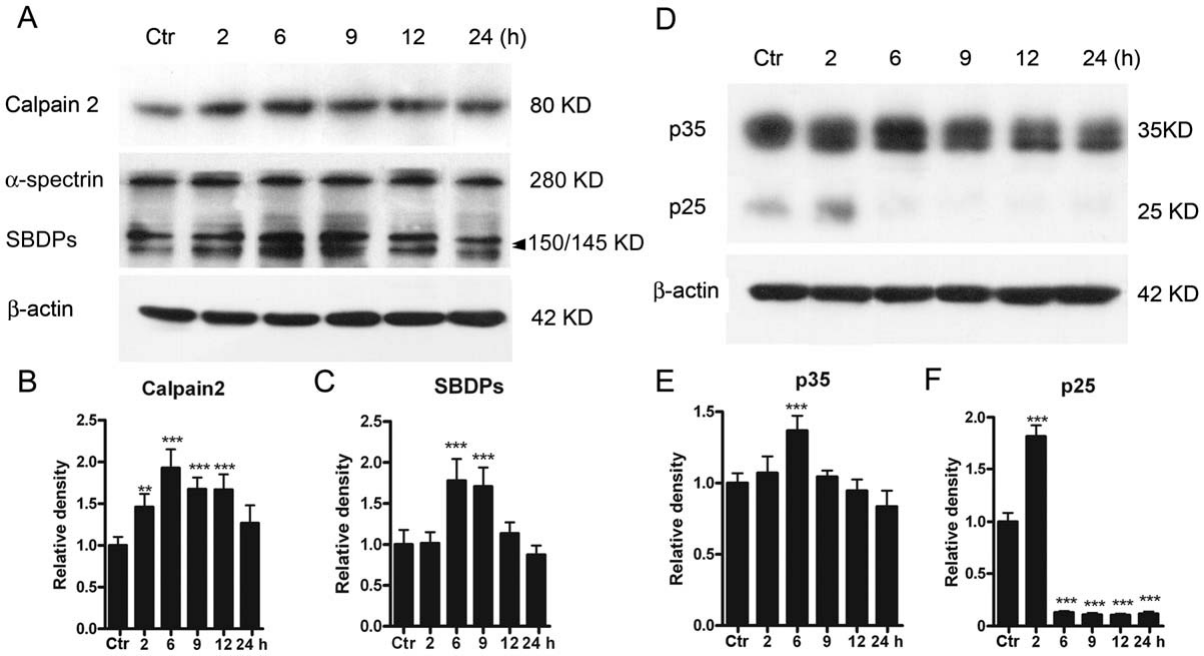
Protein levels of calpain 2, SBDPs, p35/p25 in cultured rat retinal neurons following GTs. (A) Representative immunoblots showing the changes in calpain 2 and SBDP levels in cell extracts obtained from normal (Ctr) and glutamate-treated (0.5 mM for 2, 6, 9, 12 and 24 h) groups. (B, C) Bar charts summarizing the average densitometric quantification of immunoreactive bands of calpain 2 (B) and SBDPs (C) in Ctr and glutamate-treated groups, respectively. (D) Representative immunoblots showing the changes in p35 and p25 levels in cell extracts obtained from Ctr and glutamate-treated (0.5 mM for 2, 6, 9, 12 and 24 h) groups. Note that the immunoblots for p25 were over-exposed to make them clearer. (E, F) Bar charts summarizing the average densitometric quantification of immunoreactive bands of p35 (E) and p25 (F) in Ctr and glutamate-treated groups, respectively. Note that p25 expression was sharply increased following 2 h treatment, but decreased to a very low level with longer treatments. All data are normalized to Ctr. n = 6 for each group, ** *p*<0.01, *** *p*<0.001 *vs* Ctr, one-way ANOVA.

Changes in protein levels of Cdk5 co-activators p35, p25 and calpain 2, a p35 proteolytic enzyme, were further examined. Even when the cells were challenged only by GT (2 h), the calpain 2 protein level was clearly increased to 146.1±15.5% of control (n = 6, *p*<0.01) (Figs. 3A and 3B). The protein level was further increased following GT (6 h) (193.0±22.1% of control, n = 6, *p*<0.001), and it remained at relatively higher levels [167.3±13.9% of control, n = 6, *p*<0.001 for GT (9 h) and 166.6±18.6% of control, n = 6, *p*<0.001 for GT (12 h)]. Again, the protein level tended to return to the control one following GT (24 h) (126.5±13.9% of control, n = 6, *p*>0.05).

Protein level of calpain-specific alpha-spectrin breakdown products (SBDPs) is often used to monitor the magnitude and temporal duration of calpain activation. This level was changed in parallel with the changes of calpain 2 following GTs (Fig. 3A). That is, the protein level started to increase when the cells were challenged by GT (6 h) (177.8±26.5% of control, n = 6, *p*<0.001) and further to 170.8±22.9% (n = 6, *p*<0.001) by GT (9 h) (Fig. 3C). But the level declined to the control one following GTs (12 h, 24 h). The protein level of p35 exhibited a peak (136.6±10.6% of control) with GT (6 h) (n = 6, *p*<0.001), but declined to a level comparable to the control one following GTs (9 h, 12 h, 24 h) (Figs. 3D and 3E). The change of p25 protein, a truncated form of p35, was characterized by a sharp increase (181.8±10.6% of control) with GT (2 h) (n = 6, *p*<0.001), and a subsequent large drop to a very low level following GT (6 h). The protein level remained at such low one for GTs (9 h, 12 h, 24 h) (n = 6, *p* all <0.001) (Figs. 3D and 3F).

### Calcium sources for calpain 2 activation

**Figure 4:**
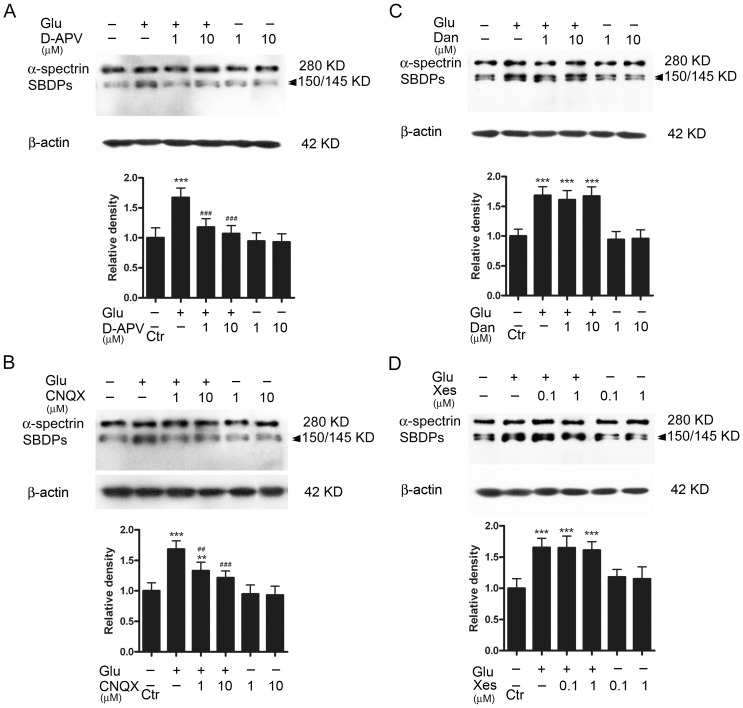
Calcium sources for the GT-induced elevation of calpain 2 protein. (A) Representative immunoblots showing the changes of SBDP levels in cell extracts obtained from normal (Ctr), glutamate (Glu)-treated (0.5 mM for 6 h) with or without addition of D-APV (1 and 10 µM), and D-APV (1 and 10 µM)-treated groups. Bar chart summarizing the average densitometric quantification of immunoreactive bands of SBDPs under different conditions. (B) Representative immunoblots showing the changes of SBDP levels in cell extracts obtained from Ctr, Glu-treated (0.5 mM for 6 h) with or without addition of CNQX (1 and 10 µM), and CNQX (1 and 10 µM)-treated groups. Bar chart summarizing the average densitometric quantification of immunoreactive bands of SBDPs under different conditions. (C) Representative immunoblots showing the changes of SBDP levels in cell extracts obtained from Ctr, Glu-treated (0.5 mM for 6 h) with or without addition of dantrolene (Dan; 1 and 10 µM), and Dan (1 and 10 µM)-treated groups. Bar chart summarizing the average densitometric quantification of immunoreactive bands of SBDPs under different conditions. (D) Representative immunoblots showing the changes of SBDP levels in cell extracts obtained from Ctr, Glu-treated (0.5 mM for 6 h) with or without addition of xestospongin (Xes; 0.1 and 1 µM), and Xes (1 and 10 µM)-treated groups. Bar chart summarizing the average densitometric quantification of immunoreactive bands of SBDPs under different conditions. All data are normalized to Ctr. n = 6 for each group, ** *p*<0.01, *** *p*<0.001 *vs* control; ^##^ *p*<0.01, ^###^ *p*<0.001 *vs* Glu alone group, one-way ANOVA.

Since calpain 2 is a calcium-dependent protease [61], we then explored Ca^2+^ source(s) for the activation of calpain 2 in glutamate neurotoxicity. There are two possible Ca^2+^ sources which could be involved in calpain 2 activation. One is the Ca^2+^ influx due to the activation of NMDARs and non-NMDARs, both of which are Ca^2+^-permeable [15]–[16], [62]. The other one is intracellular Ca^2+^ stores, from which Ca^2+^ could be released via ryanodine- and/or IP_3_-sensitive channels. In these experiments, glutamate receptor antagonists or intracellular Ca^2+^ store inhibitors were added to the culture medium 30 min prior to GT (6 h) that caused the most significant increase in calpain 2 activity (see Fig. 3B). Fig. 4A shows the effects of the addition of D-APV, a NMDAR antagonist, on the SBDP level determined by Western blotting. Following the addition of either 1 µM or 10 µM D-APV, GT (6 h) hardly increased the SBDP level (118.0±13.8% of control for 1 µM D-APV, n = 6, *p*>0.05; 107.1±13.5% of control for 10 µM D-APV, n = 6, *p*>0.05). In other words, glutamate-induced increase in SBDP level was no longer observed. As a comparison, GT (6 h) caused a considerable increase in SBDP level in the absence of D-APV (167.3±15.8% of control, n = 6, *p*<0.001 *vs* control). It is noteworthy that D-APV (either 1 µM or 10 µM) did not change the basal protein level of SBDPs (94.7±13.7% and 93.1±13.6% of control, n = 6, *p* all >0.05). The effects of the non-NMDA receptor antagonist CNQX were basically similar. In the presence of 1 µM CNQX, GT (6 h) induced a less increase in SBDP level (Fig. 4B), as compared to that obtained in the absence of CNQX. With GT (6 h) the average density of SBDP level was increased to 133.1±13.8% (n = 6, *p*<0.01 *vs* control), but much less than that obtained in the absence of CNQX (168.4±13.7%, n = 6, *p*<0.001) (Fig. 4B). When the concentration of CNQX was increased to 10 µM, the average density determined was 121.5±11.2% of control (n = 6), which was not much different from the control one (*p*>0.05) (Fig. 4B).

Effects of dantrolene, a membrane permeable intracellular ryanodine receptor antagonist, and xestospongin, a membrane permeable intracellular IP_3_ receptor antagonist, were then tested. In the presence of dantrolene of either 1 µM or 10 µM, the extent of the glutamate-induced up-regulation of SBDP protein level (161.1±15.6% of control, n = 6, for 1 µM and 167.4±15.4%, n = 6, for 10 µM) was not much changed, as compared to the level (168.6±14.6% of control) obtained with GT only but no dantrolene (Fig. 4C). The results obtained with xestospongin were similar. The average densities of SBDP proteins obtained in the presence of 0.1 µM and 1 µM xestospongin were 165.1±18.5% (n = 6) and 161.2±13.6% (n = 6) of control respectively, which were not different from that obtained in the absence of xestospongin (165.5±14.6% of control, n = 6) (*p* all >0.05) (Fig. 4D). The above results suggest that the increase in calpain 2 activation following GT (6 h) may be due to an increase in Ca^2+^ influx through NMDARs and non-NMDARs, but not due to a change in Ca^2+^ release from intracellular Ca^2+^ stores.

### Changes in p-NR2A^S1232^ protein in glutamate-treated retinal neurons

**Figure 5:**
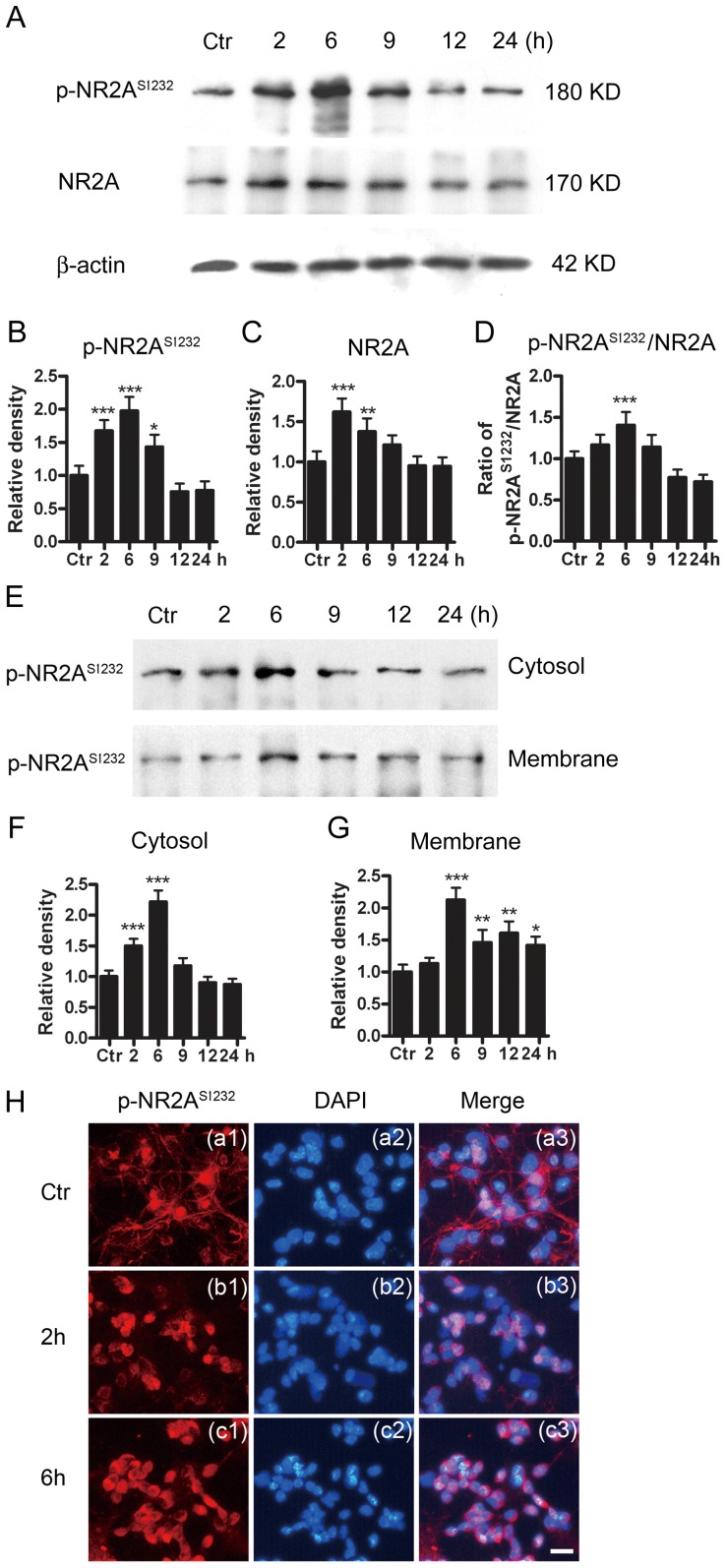
Protein level of p-NR2A^S1232^ and translocation of p-NR2A^S1232^ in cultured rat retinal neurons following GT. (A) Representative immunoblots showing the changes of p-NR2A^S1232^ and NR2A levels in cell extracts obtained from normal (Ctr) and glutamate-treated (0.5 mM for 2, 6, 9, 12 and 24 h) groups. (B, C, D) Bar chart summarizing the average densitometric quantification of immunoreactive bands of p-NR2A^S1232^, NR2A and the ratios p-NR2A^S1232^/NR2A in Ctr and glutamate-treated groups. (E) Representative immunoblots showing the changes in p-NR2A^S1232^ levels in the cytosol component and cell membrane component extracts obtained from Ctr and glutamate-treated (0.5 mM for 2, 6, 9, 12 and 24 h) groups respectively. (F, G) Bar chart summarizing the average densitometric quantification of immunoreactive bands of p-NR2A^S1232^ in the cytosol and membrane component extracts obtained from Ctr and glutamate-treated groups respectively. All data are normalized to their corresponding β-actin and then to Ctr. n = 4–6, * *p*<0.05, ** *p*<0.01, *** *p*<0.001 *vs* Ctr, one-way ANOVA. (H) Glutamate-induced translocation of p-NR2A^S1232^ in cultured retinal neurons. (a1, b1, c1) Confocal images showing immunofluorescent staining for p-NR2A^S1232^ in normal (Ctr), glutamate-treated (0.5 mM for 2 and 6 h) cells respectively. (b1, b2, c3) Counterstained images with DAPI. (c1, c2, c3) Merged images of a1 and a2, b1 and b2, c1 and c2 respectively. Note that the cell processes were shrunk following GT (2 h) and GT (6 h), and more p-NR2A^S1232^ positive signals were detected in the cytosol with GT (2 h), but in the cell membranes following GT (6 h).

Since Cdk5 phosphorylates NR2A at S1232 site in rat hippocampal CA1 neurons [45], [63], we further explored changes in NR2A and p-NR2A^S1232^ proteins following GTs for different time periods. As shown in Figs. 5A and 5B, the protein level of p-NR2A^S1232^ was greatly enhanced to 167.3±16.5% (n = 6, *p*<0.001) and 197.6±21.2% (n = 6, *p*<0.001) of control following GTs (2 h, 6 h) respectively. Following GT (9 h), the protein level declined to 143.0±18.7% of control, but still higher than the control one (n = 6, *p*<0.05). The levels became lower than the control level following GTs (12 h, 24 h) [75.5±12.5 of control for GT (12 h) and 77.5±14.1 of control for GT (24 h)]. Meanwhile, the protein level of NR2A was remarkably increased to 162.0±17.0% (n = 6, *p*<0.001) and 141.1±16.3% (n = 6, *p*<0.001) of control following GTs (2 h, 6 h) respectively, and then declined to 121.0±12.0% (n = 6, *p*>0.05), 95.1±12.1% (n = 6, *p*>0.05) and 94.4±11.2% (n = 6, *p*>0.05) of control following GTs (9 h, 12 h, 24 h) respectively (Figs. 5A and 5C). Similar changes in the ratio p-NR2A^S1232^/NR2A were observed (Figs. 5A and 5D). The ratio was increased to 140.4±16.1% of control (n = 6, *p*<0.001) for GT (6 h), but tended to decline with GTs of longer periods of time. Furthermore, subcellular distribution of p-NR2A^S1232^ was investigated in glutamate-treated retinal neurons by Western blot analysis, and some representative results are shown in Fig. 5E. The p-NR2A^S1232^ protein level in the cytosol component was increased to 149.8±11.6% (n = 4, *p*<0.001) and 211.6±18.5% of control (n = 4, *p*<0.001) for GT (2 h) and GT (6 h) respectively (Fig. 5F), but tended to return to the control level for GT (9 h), GT (12 h) and GT (24 h) (117.7±12.4%, 90.4±9.4%, 87.3±9.2% of control, respectively, n all = 4, *p*>0.05). The p-NR2A^S1232^ protein in the cell membrane component was also increased, but with a different manner. The level was increased to 212.6±19.3% of control (n = 4, *p*<0.001) for GT (6 h), but maintained at relatively higher levels for all the GTs [145.8±19.8% for GT (9 h), n = 4, *p*<0.01; 160.7±18.2% for GT (12 h), n = 4, *p*<0.01; 141.7±13.3% of control for GT (24 h), n = 4, *p*<0.05) (Fig. 5G). Such temporal shift in expression of p-NR2A^S1232^ in the cytosol and membrane suggests a translocation of this protein from the cytosol to the cell membrane.

Subcellular distribution of p-NR2A^S1232^ in cultured retinal neurons was also investigated by immunofluorescent staining (Fig. 5H). In normal (control, Ctr) retinal neurons, positive signals of p-NR2A^S1232^ were found in the somata and the cell processes (Fig. 5H a1, Ctr; a2: corresponding DAPI image of a1; a3: merged images of a1 and a2). Following GT (2 h), the cell processes were shrunk, whereas much more p-NR2A^S1232^ positive signals were detected in the somata (Fig. 5H b1). From the merged image of b1 and b2 (corresponding DAPI image of b1), it was clear that the p-NR2A^S1232^ positive signals were mostly located in the cytosol (Fig. 5H b3). With GT (6 h), positive signals for p-NR2A^S1232^ were further increased (Fig. 5H c1) and mainly seen on the cell membranes (Fig. 5H c3, merged image of c1 and c2), demonstrating a translocation of p-NR2A^S1232^ from the cytosol to the plasma membrane.

### Glutamate treatment induced changes in [Ca^2+^]_i_

**Figure 6:**
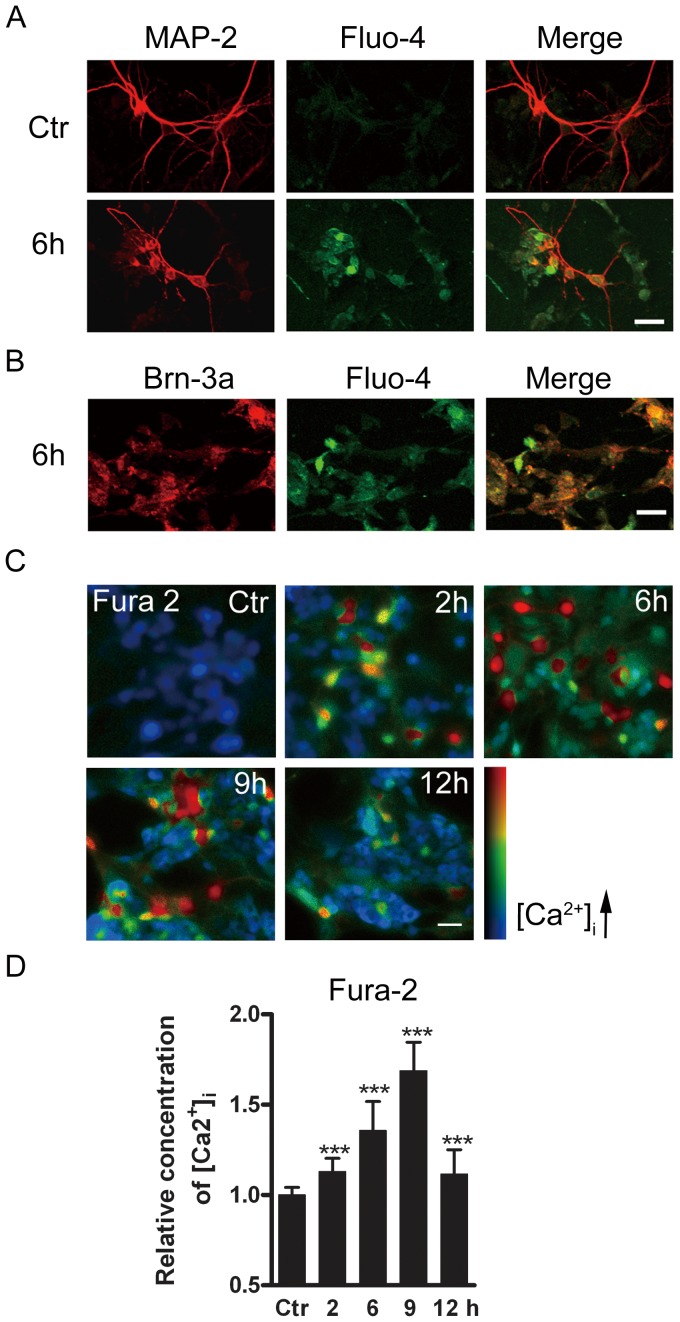
Glutamate-induced increase of [Ca^2+^]_i_ in cultured retinal neurons. (A) Confocal images showing immunofluorescent staining with MAP-2 and Fluo-4 in normal (Ctr), glutamate-treated (0.5 mM for 6 h) cells respectively. Note that the cell processes were shrunk following GT (6 h). In the merged images, it was clear that enhanced Fluo-4 signals are localized with MAP-2 positive cells. (B) Confocal images showing immunofluorescent staining for Brn-3a and Fluo-4 in the cells after GT (6 h). Note that enhanced Fluo-4 signals were observed in Brn-3a positive cells following GT (6 h). (C) Images of cultured retinal cells loaded with Fura-2 AM, taken from control cells (Ctr) and cells following GTs (2 h, 6 h, 9 h, 12 h). (D) Bar chart summarizing the changes of [Ca^2+^]_i_ in control cells (Ctr) and those following GTs (2 h, 6 h, 9 h, 12 h). [Ca^2+^]_i_, represented by the ratio of Fura-2 AM fluorescence at 340 nm and 380 nm (F340/F380), is shown in pseudocolor. The data are normalized to control. *** *p*<0.001 *vs* Ctr. Scale bar: 20 µm, for all the images.

We first tested whether retinal neurons showed changes in [Ca^2+^]_i_ following GTs. For this purpose, Fluo-4 was employed as a calcium indicator and MAP-2 as a neuronal marker. Compared to control cells (Fig. 6A, Ctr), the Fluo-4 signals in the MAP-2 positive neurons were much stronger following GT (6 h) (Fig. 6A, 6 h). From the merged image, it was clear that the change in Fluo-4 signals indeed occurred in these MAP-2 positive neurons. It was also the case for Brn-3a-positive RGCs, as shown in Fig. 6B.

We further determined how [Ca^2+^]_i_ of cultured retinal neurons was changed following GTs for different time periods by calcium imaging. Fig. 6C shows representative micrographs of retinal cell cultures following GTs (2 h, 6 h, 9 h and 12 h). Overall, [Ca^2+^]_i_ was increased with increasing times of GT in the first 9 h, then tended to decline for GT (12 h). Quantitatively, the average [Ca^2+^]_i_, represented by the ratio (F340/F380), was increased to 113.1±7.2% of control (n = 1056, *p*<0.001) for GT (2 h), and further to 135.6±16.2% (n = 920, *p*<0.001), 168.8±15.7% of control (n = 896, *p*<0.001) for GT (6 h) and GT (9 h), respectively. It declined to 111.6±13.5% of control (n = 984, *p*<0.001) for GT (12 h) (Fig. 6D).

### Regulation of protein level of p-NR2A^S1232^ by Cdk5

**Figure 7:**
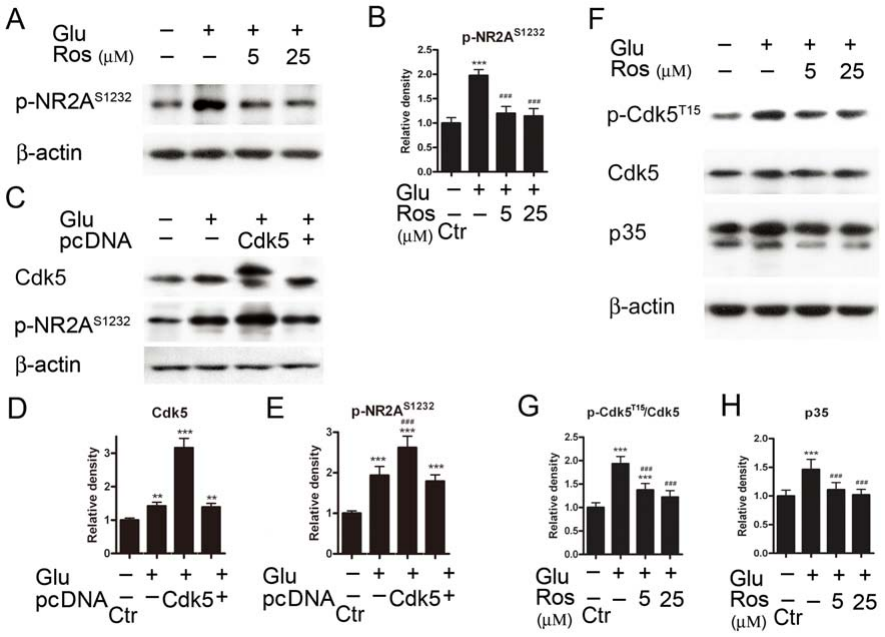
Regulation of p-NR2A^S1232^ expression by Cdk5. (A) Representative immunoblots showing the changes of p-NR2A^S1232^ levels in cell extracts obtained from Ctr and glutamate (Glu)-treated (0.5 mM for 6 h) with or without addition of roscovitine (Ros, 5 and 25 µM) groups. (B) Bar chart summarizing the average densitometric quantification of immunoreactive bands of p-NR2A^S1232^ under different conditions. (C) Representative immunoblots showing the changes in Cdk5 and p-NR2A^S1232^ levels in cell extracts obtained from Ctr and Glu-treated (0.5 mM for 6 h) groups, with or without over-expression of Cdk5. (D, E) Bar chart summarizing the average densitometric quantification of immunoreactive bands of Cdk5 and p-NR2A^S1232^ under different conditions. (F) Representative immunoblots showing the changes in p-Cdk5^T15^,Cdk5 and p35 levels in cell extracts obtained from Ctr and glutamate (Glu)-treated (0.5 mM for 6 h) groups, with or without addition of roscovitine (Ros, 5 and 25 µM). (G, H) Bar charts summarizing the average densitometric quantification of immunoreactive bands of p-Cdk5^T15^/Cdk5 (G) and p35 (H) under different conditions, respectively. All data are normalized to Ctr. n = 6, ** *p*<0.01 and *** *p*<0.001 *vs* control; ^###^ *p*<0.001 *vs* Glu-treated group, one-way ANOVA.

To explore whether the elevated protein level of p-NR2A^S1232^ following GT (<6 h) may be mediated by an activation of Cdk5, roscovitine was added to the culture medium 30 min prior to GT (6 h). As shown in Figs. 7A and 7B, roscovitine of 5 µM almost blocked the glutamate-induced upregulation of p-NR2A^S1232^ protein, with an average density of p-NR2A^S1232^ proteins being 120.0±13.9% of control (n = 6). This density level was not different from the control one (*p*>0.05), but much lower than that obtained in the absence of roscovitine (*p*<0.001). With 25 µM roscovitine, the density was reduced (114.3±15.5% of control, n = 6, *p*<0.05 *vs* control). On the other hand, GT (6 h) caused a significant increase in Cdk5 protein level in the cells with Cdk5 being over-expressed, with an average density being 316.2±28.0% of control (n = 6, *p*<0.001), much higher than that obtained from normal cells (142.0±11.0% of control, n = 6, *p*<0.01) or vector expressed cells (139.4±10.5% of control, n = 6, *p*<0.01) (Fig. 7C, 7D). Meanwhile, GT (6 h) caused an even larger increase in p-NR2A^S1232^ protein in the cells with Cdk5 being over-expressed, with an average density being 262.4±28.0% of control (n = 6), much higher than that (193.4±22.1% of control) obtained from normal cells (*p*<0.001) (Fig. 7E). Furthermore, an examination of effects of roscovitine on protein levels of Cdk5, p-Cdk5 and p35 following GT (6 h) (Fig. 7F) revealed that roscovitine reduced the glutamate-induced upregulation of both the ratio p-Cdk5^T15^/Cdk5 and p35 protein level. The ratio p-Cdk5^T15^/Cdk5 was 137.5±13.7% of control with 5 µM roscovitine treatment (n = 6, *p*<0.001), which was significantly lower than that obtained without roscovitine treatment (193.4±15.6% of control, n = 6, *p*<0.001) (Fig. 7G). When the roscovitine concentration was increased to 25 µM, similar reduction was seen (122.1±13.8% of control, n = 6, *p*<0.001). Similar results were observed concerning the effect of roscovitine treatment on p35 protein level. That is, the average density of p35 was reduced from 146.2±17.8% of control (n = 6) in the GT (6 h) alone group to 110.0±12.4% (for 5 µM roscovitine, n = 6, *p*<0.001) and 101.9±9.6% of control (for 25 µM roscovitine, n = 6, *p*<0.001), respectively (Fig. 7H).

## Discussion

Considerable evidence has demonstrated that over-activation of functional NMDARs and non-NMDARs, both of which are expressed on RGCs, may play a crucial role in RGC death, occurring in glaucoma, diabetic retinopathy, retinal ischemia, and other retinal diseases [8], [10]–[11], [17], [64]. RGCs in vitro and in vivo are highly vulnerable to glutamate neurotoxicity [65], [66]. Consistent with these reports, using cultured mixed retinal neurons, about 64% of which were RGCs, we showed that GT (24 h) caused a robust decrease in cell viability and significant cell apoptosis, with most of the apoptotic cells (83%) being RGCs (Fig. 1D, 1F). This result is different from the work of Ullian *et al.* [67], which showed that the exposure of glutamate or NMDA of high concentrations did not cause the death of rat purified RGCs and mixed cells. A possible explanation for the inconsistence could be that in the work of Ullian *et al.* [67], all the data presented were obtained when cultured cells were exposed to 0.5 mM glutamate only for 1 h (though it was claimed that it was also the case even after prolonged exposure for 24 h), but the results reported in the present work were obtained when the cells were treated with glutamate for 6–24 h. Actually, under our experimental conditions we found no change in morphology of cultured cells following GT (1 h) (data not shown). It should be also noted that in the work of Ullian *et al.* whether or not the cells underwent apoptosis was not examined.

Cdk5 plays pleiotropic roles in both neuronal physiological functions and degeneration of neurons [28], [37], [39]–[41], [46], [68]–[70]. In the retina, Cdk5 activation is involved in axotomy-induced RGC death [71] and intraocular hypertension-induced RGC apoptosis [44]. The increase in protein levels of both Cdk5 and p-Cdk5^T15^ observed following GTs suggests an increased activation of Cdk5 [57]. The enhanced Cdk5 activation evidently contributed to GT-induced cell death and apoptosis because administration of roscovitine largely blocked GT-induced decrease of cell viability (Fig. 2E) and reduced the number of apoptotic cells (Fig. 2F). The upregulation of Cdk5 may be a result of the elevated protein level of the Cdk5 co-activator p35, as suggested by similar changes in p35 and Cdk5 protein levels as a function of glutamate exposure time (compare Fig. 3E with Fig. 2C).

Cell apoptosis is controlled by several proteases [72]–[75]. In addition to caspases, which are cysteine proteases, calpain plays an important role in cell apoptosis in various neuronal tissues [76]. In the retina, calpain is present in RGCs, and its activation is detected in the ganglion cell layer in retinal explants after axotomy [77] and in a rat experimental glaucoma model [78]. Calpain inhibitors are shown to protect RGCs from apoptosis induced by axotomy [77]. Ca^2+^-induced activation of calpain also leads to photoreceptor cell apoptosis [61]. In addition, calpain-dependent proteolysis of alpha-spectrin, tau, and p35 was observed in the retina after ocular hypertension [79]. Calpain 2 is one of the major calpain isoforms and its mRNA is twelve times more than calpain 1 in retinas [80]. Moreover, calpain 2 is more sensitive to Ca^2+^ than calpain 1 [81]. In the present work, GT induced an increase in protein levels of calpain 2 and SBDPs. It has been shown that sustained expression of SBDPs could further strengthen the activation of calpain 2 [82], [83].

Since calpain could cleave proteolytically p35 into p25, it may be expected that the protein level of p25 was elevated following GT (Fig. 3F). Increased cleavage was detected in rat cultured neurons undergoing cell death [34]. It is known that p25 activates Cdk5 more efficiently and results in deleterious effects on neurons in many neurodegenerative diseases [33]–[35], [84]. The increased p25 protein may further promote Cdk5 expression and consequential cell death and/or apoptosis. It was of interest that the increase in p25 protein was transient, while the increase in calpain 2 level was rather sustained, lasting for much longer time (compare Fig. 3B with Fig. 3F). We speculate that upregulated phosphorylation of p35 by elevated activation of Cdk5 may suppress both proteasome-mediated degradation of p35 and calpain-mediated cleavage of p35 [84]–[86]. The activation of calpain 2 following GT was due to an increased Ca^2+^ influx through both NMDARs and non-NMDARs, but not related to intracellular Ca^2+^ stores. This result is consistent with that obtained in cultured rat hippocampal neurons [62]. Indeed, calpain signaling could be activated by various pathways that elevate [Ca^2+^]_i_. In a study performed by Das et al. [87], they found that a 24 h ionomycin (IMN) or interferon-gamma (IFN-gamma) exposure induced a significant increase in [Ca^2+^]_i_, thereby activating calpain signaling. It was suggested that the elevation of [Ca^2+^]_i_ may be due to Ca^2+^ influx and/or Ca^2+^ release from intracellular stores. In the present work, we demonstrated that glutamate exposure activated calpain signaling by increasing Ca^2+^ influx, but not Ca^2+^ release from intracellular stores. It should be indicated, however, because activation of calpain is Ca^2+^-dependent, any treatment that causes intracellular Ca^2+^ overload, as Das et al did, could induce the activation of calpain signaling, thereby leading to RGC apoptotic death [88]. Therefore, our result is not contradictory to that of Das et al.

How is Cdk5 involved in RGC apoptosis in glutamate neurotoxicity? Among others, a possibility that our results support may be described as follows. Cdk5 phosphorylates NR2A-containing NMDARs at site 1232, which is crucial for glutamate-induced retinal cell injury [44]. This event is followed by a translocation of p-NR2A^S1232^ from the cytosol to the cell membrane, thus enhancing the expression of functional NMDARs in the cell membrane and boosting the glutamate-induced increase of [Ca^2+^]_i_ (Fig. 6). The evidence in favor of this possibility is twofold. First, roscovitine inhibited the glutamate-induced increase of p-NR2A^S1232^ protein in the cells, but over-expression of Cdk5 further boosted it (Fig. 7), suggesting the involvement of Cdk5 in the elevation of p-NR2A^S1232^ protein. Secondly, translocation of p-NR2A^S1232^ from the cytosol to the plasma membrane was indeed shown. It was noteworthy that GT also induced a robust increase in protein level of the NR2A subunit. Such increased NR2A protein level may contribute to the elevation of p-NR2A^S1232^ level as well. It should be noted that, like the changes in calpain 2, Cdk5 and p-NR2A^S1232^ protein levels, [Ca^2+^]_i_ was steadily increased following GTs (≤9 h), but tended to decline following longer (>9 h) GTs. This suggests that the increase in [Ca^2+^]_i_ due to the translocation of p-NR2A^S1232^ from the cytosol to the plasma membrane may occur only in the early stage of glutamate-induced injury under our cultured condition.

It seems a paradox that the protein levels of Cdk5, p-Cdk5^T15^ and [Ca^2+^]_i_ almost returned to the control ones when the cells were challenged with GT (24 h), but cell apoptosis was still clearly seen (Fig. 1). A possible explanation is that apoptosis is a cascade of cellular events, such as activation of the mitochondrial permeability transition, release of pro-apoptotic proteins, and activation of poly(ADP-ribose) polymerase-1 and so on [89], [90], leading to programmed self-destruction of a cell [91]–[93]. Once this cascade is triggered by some factor(s), it will go on following a pre-programmed procedure no matter whether the triggering factor(s) still exists or not. In our case, the elevated levels of Cdk5 and [Ca^2+^]_i_, which occurred at the early phase [GT (≤9 h)], triggered cell apoptosis in cultured retinal neurons, and the apoptosis process could keep on even though the protein levels of Cdk5 and [Ca^2+^]_i_ have somewhat returned to the normal ones. In this context, it has been suggested that cell injury depends more on how or where calcium enters the cell rather than on how much enters [89], [94], [95].

In summary, our results suggest a possible mechanism for glutamate-induced injury of retinal neurons as follows. Over-activation of both NMDARs and non-NMDARs induced by excessive glutamate leads to an increase in intracellular Ca^2+^ levels, thus enhancing the expression of p-NR2A^S1232^, especially on the membrane, through a calpain/p35-p25/Cdk5 signaling pathway. The enhanced expression of functional NMDARs will in turn render the cells with more Ca^2+^ overload, thereby further aggravating the cell injury. All these changes occur at the early phase of glutamate-induced cell injury, which is followed by a cascade of cellular events, resulting in programmed cell death.
